# Cognitive impairment in manic bipolar patients: important, understated, significant aspects

**DOI:** 10.1186/s12991-015-0080-0

**Published:** 2015-11-25

**Authors:** Mădălina Vrabie, Victor Marinescu, Anca Talaşman, Oana Tăutu, Eduard Drima, Ioana Micluţia

**Affiliations:** University of Medicine and Pharmacy “Iuliu Hatieganu” Cluj-Napoca, Cluj-Napoca, Romania; 7th Ward, Clinical Hospital of Psychiatry “Al. Obregia” Bucharest, Bucharest, Romania; University of Medicine and Pharmacy “Carol Davila” Bucharest, Bucharest, Romania; 9th Ward, Clinical Hospital of Psychiatry “Al. Obregia” Bucharest, Bucharest, Romania; Department of Cardiology, Clinical Emergency Hospital Bucharest, Bucharest, Romania; Faculty of Medicine and Pharmacy, “Danubius” University, Galati, Romania; Clinical Hospital of Psychiatry “Elisabeta Doamna” Galati, Galati, Romania; Second Psychiatric Clinic, Emergency County Hospital Cluj-Napoca, Cluj-Napoca, Romania

**Keywords:** Bipolar, Cognitive, Mania

## Abstract

**Background:**

Bipolar disorder is a chronic mood disorder with episodic progress and high relapse rate. Growing evidence suggests that individuals with bipolar disorder display cognitive impairment which persists even throughout periods of symptom's remission.

**Method:**

137 bipolar patients met the inclusion criteria (depressive episode: DSM-IV-TR criteria for major depressive episode, HAMD score ≥17; manic/hypomanic episode: DSM-IV-TR criteria for manic/hypomanic episode, YMRS score ≥12, euthymic: 6 months of remission, HAMD score ≤8, YMRS score ≤6; and mixed: DSM-IV-TR criteria for mixed episode, HAMD score >8 and YMRS score >6) and were therefore enrolled in the study. Patients were free of psychotic symptoms (hallucinations/delusions) at the moment of testing. Control group consisted of 62 healthy subjects without history of neurological and/or psychiatric disorder. Cognitive battery has been applied in order to assess verbal memory, working memory, psychomotor speed, verbal fluency, attention and speed of information processing, and executive function. Following data were collected: demographics, psychiatric history, age of illness onset; current and previous treatment (including hospitalizations). Cognitive deficits were assessed in bipolar patients experiencing manic, depressive, mixed episodes or who were euthymic in mood. Results were compared between the subgroups and with healthy individuals. The association of impaired cognition with illness course was analyzed.

**Results:**

Bipolar patients showed cognitive deficits in all evaluated domains when compared to controls. The lowest scores were obtained for the verbal fluency test. After adjusting for current episode, manic subgroup showed greater cognitive impairment in verbal and working memory, executive function/reasoning and problem solving, compared to depressive, mixed, and euthymic subgroup. Low-neurocognitive performance was directly associated with a predominance of manic episodes and severe course of bipolar illness. An increased number of past manic episodes was the strongest correlated event with the poorest outcomes in verbal memory testing. Other factors correlated with poor verbal memory scores in manic subgroup were age at illness onset (positive correlation), illness length, and hospitalizations (negative correlations).

**Conclusions:**

Bipolar patients showed cognitive deficits regardless of the phase of illness. Subjects experiencing a manic episode displayed higher deficits in verbal and working memory, executive function/reasoning, and problem solving. Severe course of illness also showed significant contribution in terms of cognitive impairment.

## Background

The bipolar disorder is a chronic disorder with a high relapse rate, significant general disability, personal and social burden, and psychosocial impairment. These deficits often persist despite pharmacotherapy [1]. Bipolar patients often suffer from “debilitating” cognitive impairment in different stages of the disease [2]. Areas such as sustained attention [3], memory [4] and executive function [5] are involved. These deficits are present during the acute phase of illness and persist throughout periods of euthymia [6]. The mood changes in acute episodes have a clear impact on cognitive functioning [7]. The cognitive deterioration is often considered as a basic characteristic of this psychiatric disorder [2]. Deficits in cognitive function are both transitory (acute phase of illness) and persistent (chronic/residual symptoms) [7].

The disorder’s severity seems to have an important role in terms of cognitive deficits [4, 8]. Several studies [6, 9–12] show that chronic patients or patients having a history of multiple affective episodes which required re-hospitalizations, suffer from more significant cognitive deficits. Cognitive impairment has serious consequences for patients and caregivers, by impacting on the quality of family life [13]. Martinez-Aran et al. showed that there is a correlation between the impairment of verbal memory and illness length, number of past manic episodes, and number of psychiatric hospitalizations [6].

The objective of this study is to evaluate the cognitive deterioration in bipolar disorder and to identify the correlation between cognitive impairment and the course of bipolar illness (characterized by illness length, onset age, number of hospitalizations, and predominant polarity of previous episodes).

## Method

### Hypothesis

Patients with bipolar disorder show significant cognitive deficits that often are underestimated and underdiagnosed. These deficits occur in both acute episodes and in euthymia. In our view, a complex and thorough evaluation of patients with bipolar disorder should include the assessment of neurocognitive functioning (both at the onset and during the disease). The cognitive impairment must be considered as a core feature of the bipolar disorder, which finally influences remission and overall functioning. We believe that the severe course of bipolar disorder (characterized by early onset illness, longer illness length, and a higher number of hospitalizations) negatively affects the bipolar patients’ cognitive status. A higher number of past manic episodes seems to exacerbate cognitive impairment more than the presence of a higher number of past depressive episodes.

### Study population

The target and accessible population is represented by patients with Bipolar I Disorder, recorded at ‘Al. Obregia’ Clinical Hospital of Psychiatry Bucharest, Romania, during January 1st, 2012–January 1st, 2015. This observational study included two groups of subjects: the bipolar group—with 137 patients, and the control group—with 62 healthy subjects. The bipolar group consists of four subgroups: 54 manic patients, 32 depressive patients, 30 euthymic patients, and 21 patients with mixed episodes. All patients were clinically assessed, and cognitive testing was performed at baseline and follow-up. Preliminary data presented in this study include baseline assessment measures. These results will be further used in comparison with data outcomes from follow-up assessments. The control group (*n* = 62 healthy subjects) was recruited through local advertising. Both patients and controls who were included in this study met all inclusion criteria and signed the informed consent approved by the Ethics Committee from the University of Medicine and Pharmacy “Iuliu Hatieganu” Cluj-Napoca, Romania.

### The inclusion criteria

Patients were eligible to be included in the study if they met all the inclusion criteria. Both male and female patients aged 18–50, with a diagnosis of Bipolar I Disorder and at least 5 years of formal education (educational status is given as duration of education in years) were considered eligible. The patient's subgroups were characterized by the following requirements: depressive episode (DSM-IV-TR criteria for major depressive episode, HAMD ≥17), manic/hypomanic episode (DSM-IV-TR criteria for manic/hypomanic episode, YMRS ≥12), euthymia (at least 6 months of remission, HAMD = 8, YMRS = 6), and mixed (DSM-IV-TR criteria for mixed episode, HAMD score >8 and YMRS score >6) [14]. Prior to the full set of the cognitive battery, patients were required to retain at least five words over five trials on the ‘List Learning’ (subtest of the Brief Assessment of Cognition in Schizophrenia) [15]. Those patients with low performance at this test were excluded from the study due to the fact that the neurocognitive battery would have not been valid. No requirements in terms of psychiatric medication were made in the inclusion criteria. In order for the tests to be considered reliable, subjects had to prove a level of understanding which would allow them to perform all the examinations required by the protocol.

### The exclusion criteria

Hallucinations and disruptive behavior at the moment of examination; low understanding of psychometric tasks; substance abuse or dependence (except nicotine and caffeine) in the present or in the past; past or current somatic and/or psychiatric comorbidities which may affect cognitive status of the bipolar patients: Alzheimer’s Disease, other dementias, retardation, stroke; lengthy surgical intervention with general anesthesia; head trauma with/without loss of consciousness; massive bleeding; voluntary/involuntary poisoning with carbon monoxide and other; electroconvulsive therapy in the last year; somatic treatment with drugs known to affect cognitive status (cortisol, antihistamines, etc.).

### The control sample

Healthy subjects aged 18–50 years; at least 5 years of formal education; no past or current psychiatric or neurological disorders; absence of somatic and/or psychiatric comorbidities which may affect their cognitive status; no relatives with bipolar disorder or psychotic disorder; no substance abuse or dependence (except nicotine and caffeine) in the present or in the past. Control subjects were matched with bipolar patients in terms of age, gender, and education level.

Demographic and clinical data were systematically obtained and included gender, age, years of education, current diagnosis, past psychiatric history, illness length, and illness onset age (defined as the age when the subjects first experienced an episode of illness regardless of the polarity). YMRS [16] and HAMD [17] were performed in order to include the subjects in bipolar subgroups.

The neuropsychological assessment: Romanian language version of the following tests: ‘list learning,’ ‘digit sequencing task,’ ‘token motor task,’ ‘category instances (semantic fluency)’ and ‘controlled oral word association test (letter fluency),’ ‘symbol coding,’ and ‘tower of London.’ The key features of these tests were brief administration and scoring time, portability, repeatability, and availability of alternate forms; these could also be accepted for the investigation of cognitive domains in bipolar disorder even from the study’s beginning. The domains of cognitive function, which are assessed by these tests, are those found to be consistently impaired and related to outcome in bipolar disorder (e.g., verbal memory, working memory, psychomotor speed, attention and speed of information processing, executive function/reasoning and problem solving, and verbal fluency). The cognitive domains assessed were verbal memory—“list learning,” working memory—“digit sequencing task,” psychomotor speed—“token motor task,” verbal fluency (processing speed)—“category instances (semantic fluency)” and “controlled oral word association test (letter fluency),” attention and speed of information processing—“symbol coding,” and executive functions/reasoning and problem solving—“tower of London” [15, 18, 19]. The assessment of cognitive function was performed by a psychiatrist trained and certified for neurocognitive assessment. All subjects were free of psychotic symptoms at the moment of testing.

Comparison in cognitive function between the bipolar group and control group was performed. Afterward, cognitive function was compared between the subgroups of bipolar patients: manic subgroup vs. depressive subgroup vs. euthymic subgroup vs. mixed subgroup. In the lead of all these steps, the analysis emphasized two important correlations: one between cognitive function and predominant polarity and another one between cognitive function and previous course of the illness (illness length, onset age, and number of hospitalizations). The bipolar group was divided (according to the main polarity of the previous episodes) in two subgroups: one with previous mainly manic episodes (number of previous manic episodes exceeded the number of previous depressive episodes) and another one with previous mainly depressive episodes (the number of previous depressive episodes was greater than the number of previous manic episodes). The cognitive assessment was performed in a cross-sectional manner, while the disorder’s evolution (from onset to present) was studied retrospectively (complete data related to the history of medication and the periods when the patients were untreated were not available). Therefore, this study was conducted in a naturalistic treatment setting. No special analysis has endorsed to medication. Findings regarding the correlation between medication status and the cognitive function should be considered exploratory, and might require further investigation and do not make the object of this study.

The statistical analysis was performed with IBM SPSS Statistics 20.0 software at a significance level of *p* ≤ 0.05. A descriptive analysis (means, medians, standard deviations, and range for continuous data and frequency analysis for categorical data) was performed for all the target variables. Kolmogorov–Smirnov test was used to analyze continuous data distribution, according to which appropriate tests were further applied: ANOVA (with Bonferroni post Hoc Test) or Kruskal–Wallis test for differences between means of three independent groups. Chi square test was used to analyze differences between categorical data. The correlations were based on Pearson’s correlation coefficient or Spearman’s correlation coefficient.

## Results

Socio-demographic characteristics of the samples are presented in Table 1. There can be seen a female predominance in the control group (F 59.7 % vs. M 40.3 %) as well as in the bipolar group (F 62.8 % vs. M 37.2 %). There were no significant differences between the two study groups regarding age [*t* = −0.51; 95 % CI: (−2.81; 1.66); *p* = 0.613], gender, and education level [*t* = 1.09; 95 % CI (−0.32; 1.09); *p* = 0.164]. The mean age at onset was 24 years, and the mean length of illness was of 18 years. The number of hospitalizations had a median value of 11. Regarding the medication at the time of cognitive evaluation, we present the following figures: 88 patients (64.23 %) received antipsychotics, 90 (65.69 %) patients received benzodiazepines, 60 (43.79 %) patients were both on antipsychotics and benzodiazepines, and 50 (36.49 %) patients have been treated with antidepressants.

**Table 1:** Socio-demographic and illness characteristics of the study groups

	Bipolar group *N* = 137	Control group *N* = 62
Age (Y)^a^	39.01 ± 7.18	38.44 ± 7.45
Education (Y)^a^	13.95 ± 2.17	14.34 ± 2.39
Age at illness onset (Y)^a^	24 (19–28)	–
Illness length (Y)^a^	18 (2–30)	–
Hospitalizations^a^ (*N*)	11 (3–23)	–
Gender		
Female	86 (62.8)	37 (59.7)
Male	51 (37.2)	25 (40.3)
Occupational status		
Employed	72 (52.6)	49 (79)
Unemployed	32 (23.4)	13 (21)
Retired (medical)	33 (24.1)	00 (0)

*Y* years, *N* numbers

^a^Values are presented as mean ± standard deviation for parametric scale variables and mean (range) for nonparametric scale variables

### Cognitive function scores across study groups

As compared to the control group, bipolar patients reached significantly lower scores in all tests, namely verbal memory [mean differences = 8.2; *t* = 13.95; 95 % CI = (7.04; 9.36), *p* < 0.0001], working memory (median differences = 9; *Z* = −9.85; *p* < 0.0001), psychomotor speed (median differences = 16; *Z* = −10.85; *p* < 0.0001), verbal fluency [mean differences = 20.25; *t* = 16.44; 95 % CI = (17.82; 22.68), *p* < 0.0001], attention and speed of information processing (median differences = 17.5; *Z* = −11.21; *p* < 0.0001), and executive function/reasoning and problem solving (median differences = 8; *Z* = −11.28; *p* < 0.0001) (Table 2).

**Table 2:** Cognitive function scores across study groups

	Bipolar group *N* = 137	Control group *N* = 62	*p*
Verbal memory	40.53 ± 4.16	48.73 ± 3.02	<0.0001*
Working memory	18 (10–28)	27 (24–29)	<0.0001**
Psychomotor speed	54 (42–74)	70 (64–72)	<0.0001**
Verbal fluency	46.02 ± 9.52	66.27 ± 2.69	<0.0001*
Attention and speed of information processing	42 (30–58)	59.5 (54–63)	<0.0001**
Executive function/reasoning and problem solving	13 (4–19)	21 (17–22)	<0.0001**

Values are presented as mean ± standard deviation for continuous parametric data and mean (range) for nonparametric continuous data

*NS* no statistical significant (*p* ≥ 0.05)

* Independent samples *t* test; ** Mann–Whitney *U* test

No significant gender differences were found in terms of cognitive function.

### Cognitive function scores across study subgroups

Most bipolar patients in the study group were represented by manic patients. [manic: 54 (39.4 %), depressive 32 (23.4 %), euthymic: 30 (21.9 %), and mixed 21 (15.3 %)]. There were no significant differences between the four subgroups regarding the age, gender, and education level, these subgroups being statistically similar. With regard to the occupational status, while most manic and depressive patients were in their majority either unemployed or retired, patients with mixed episodes and euthymia were mostly employed (Table 3).

**Table 3:** Socio-demographic characteristics of study subgroups

	Manic subgroup *N* = 54	Depressive subgroup *N* = 32	Euthymic subgroup *N* = 30	Mixed subgroup *N* = 21	*p*
Age (Y)	39.33 ± 6.94	39.56 ± 7.67	37.97 ± 7.17	38.81 ± 7.42	NS*
Gender					
Male	21 (41.2)	8 (15.7)	13 (25.5)	9 (17.6)	NS**
Female	33 (38.4)	24 (27.9)	17 (19.8)	12 (14)	NS**
Education (Y)	15 (10–17)	12 (10–17)	15 (10–16)	15 (10–17)	NS***
Occupational status					
Unemployed	14 (43.8)	8 (25)	0 (0)	10 (31.2)	0.001**
Employed	25 (34.7)	13 (18.1)	24 (33.3)	10 (13.9)	0.001**
Retired (medical)	15 (45.5)	11 (33.3)	6 (18.2)	1 (3)	0.001**

Values are presented as mean ± SD for continuous parametric data, median (range) for continuous nonparametric data and absolute number (percent) for categorical data

*Y* years, *NS* no statistical significant (*p* ≥ 0.05)

* ANOVA; ** Chi square test; *** Kruskal–Wallis test

The lowest scores on verbal memory test were registered in manic patients. Subjects with manic and mixed episodes obtained low scores in working memory, executive function/reasoning, and problem solving tests. Depressive episodes were associated with lower scores in psychomotor speed and verbal fluency tests. Patients with mixed episodes had a poor measurement outcome in terms of attention and speed of information processing tests (Table 4).

**Table 4:** Cognitive function scores across study subgroups

	Manic subgroup *N* = 54	Depressive subgroup *N* = 32	Euthymic subgroup *N* = 30	Mixed subgroup *N* = 21	p*
Verbal memory	37 (30–46)	41 (38–47)	45 (39–47)	40 (37–45)	<0.0001
Working memory	14 (10–28)	20.5 (10–28)	25 (14–27)	14 (11–20)	<0.0001
Psychomotor speed	54 (47–74)	48 (44–58)	58 (54–62)	46 (42–68)	<0.0001
Verbal fluency	44.5 (34–67)	34 (30–55)	56 (50–62)	41 (40–46)	<0.0001
Attention and speed of information processing	42 (39–58)	41 (39–48)	48 (43–52)	40 (30–44)	<0.0001
Executive function/reasoning and problem solving	11.5 (4–18)	14 (11–19)	16 (12–17)	11 (7–15)	<0.0001

Values are presented as median (range) for continuous nonparametric data

*NS* no statistical significant (*p* ≥ 0.05)

* Kruskal–Wallis test

The youngest age at illness onset was recorded in the manic subgroup (median = 22-year old). There were no significant differences between the four subgroups of bipolar patients in terms of illness length. The smallest number of hospitalizations was noted in euthymic patients, which was significantly lower than the number of hospitalizations recorded in patients with manic (mean difference = −3.38), depressive (mean difference = −4.04), and mixed (mean difference = −4.11) episodes. These last three subgroups registered statistically similar numbers of psychiatric hospitalizations (Table 5).

**Table 5:** Illness characteristics of the study groups

	Manic subgroup *N* = 54	Depressive subgroup *N* = 32	Euthymic subgroup *N* = 30	Mixed subgroup *N* = 21	*p*
Age at illness onset (Y)	22 (19–28)	23 (20–27)	25.5 (24–28)	23 (20–28)	<0.0001*
Illness length (Y)	16.59 ± 7.59	17.09 ± 8.43	12.3 ± 6.86	15.29 ± 7.27	NS**
Hospitalizations (*N*)	11.65 ± 4.97	12.31 ± 4.73	8.27 ± 4.10	12.38 ± 6.37	0.005**

Values are presented as mean ± SD for continuous parametric data, median (range) for continuous nonparametric data

*Y* years, *N* numbers, *NS* no statistical significant (*p* ≥ 0.05)

* Kruskal–Wallis test; ** ANOVA

### Correlations between cognitive functions and illness characteristics

The strongest highlighted correlations within the bipolar group were between the age at illness onset and verbal fluency scores (*r*_s_ = 0.534; *r*_s_^2^ = 0.285; *p* < 0.0001), illness length and psychomotor speed scores (*r*_s_ = −0.599; *r*_s_^2^ = 0.359; *p* < 0.0001), hospitalizations number and verbal memory scores (*r*_s_ = −0.694; *r*_s_^2^ = 0.482; *p* < 0.0001), and number of hospitalizations and working memory scores (*r*_s_ = −0.556; *r*_s_^2^ = 0.482; *p* < 0.0001).

After adjusting for the current polarity of episodes, robust correlations are as follows:

In manic subgroup, verbal memory scores correlated with age at illness onset (*r*_s_ = 0.454; *r*_s_^2^ = 0.206; *p* = 0.001), illness length (*r*_s_ = −0.681; *r*_s_^2^ = 0.464; *p* < 0.0001), and number of hospitalizations (*r*_s_ = −0.839; *r*_s_^2^ = 0.704; *p* < 0.0001).

In the depressive subgroup, the strongest correlations were between age at illness onset and verbal fluency scores (*r*_s_ = 0.740; *r*_s_^2^ = 0.548; *p* < 0.0001), illness length and working memory scores (*r*_s_ = −0.892; *r*_s_^2^ = 0.796; *p* < 0.0001), and number of hospitalizations and working memory scores (*r*_s_ = −0.861; *r*_s_^2^ = 0.741; *p* < 0.0001).

In the euthymic subgroup, attention and speed of information processing scores were proved significant correlation with illness length (*r*_s_ = −0.695; *r*_s_^2^ = 0.483; *p* < 0.0001) and number of hospitalizations (*r*_s_ = −0.755; *r*_s_^2^ = 0.570; *p* < 0.0001).

In the mixed episodes subgroup, attention and speed of information processing scores were the strongest correlated with illness length (*r*_s_ = −0.907; *r*_s_^2^ = 0.823; *p* < 0.0001) and hospitalizations number (*r*_s_ = −0.912; *r*_s_^2^ = 0.832; *p* < 0.0001).

### Cognitive function scores across bipolar patients with previous mainly manic and previous mainly depressive episodes

The majority of bipolar patients (96, 70.1 %) had a predominance of manic episodes throughout the course of illness. The subgroup with a predominance of previous manic episodes had significantly lower scores than the subgroup with previous mainly depressive episodes on the verbal memory testing [mean difference = −4.233, *t* = −7.05, 95 % CI = (−5.42; −3.04)], working memory testing (median difference = −7, *Z* = −3.87), attention and speed of informational processing testing (median difference = −2, *Z* = −2.58), and executive function/reasoning and problem solving testing [mean difference = −2.2, *t* = −3.60, 95 % CI = (−3.41; −0.99)] (Table 6).

**Table 6:** Cognitive function scores across bipolar patients with previous mainly manic and previous mainly depressive episodes

	Previous episode polarity		*p*
	Mainly manic *N* = 96	Mainly depressive *N* = 41	
Verbal memory	39.26 ± 4.00	43.49 ± 2.81	<0.0001*
Working memory	17 (10–28)	24 (11–27)	<0.0001**
Psychomotor speed	54.09 ± 5.65	53.05 ± 6.63	NS*
Verbal fluency	43.5 (31–67)	50 (30–61)	NS**
Attention and speed of information processing	42 (30–50)	44 (34–58)	0.010**
Executive function/reasoning and problem solving	12.17 ± 3.52	14.37 ± 2.58	<0.0001*

Values are presented as mean ± SD for continuous parametric data, median (range) for continuous nonparametric data

*NS* no statistical significant (*p* ≥ 0.05)

* Kruskal–Wallis test; ** ANOVA

The predominance of previous manic episodes was correlated with lower scores in working memory (*r*_s_ = 0.332; *r*_s_^2^ = 0.110; *p* < 0.0001), attention and speed of information processing (*r*_s_ = 0.222; *r*_s_^2^ = 0.049; *p* = 0.009), executive function/reasoning and problem solving (*r*_s_ = 0.289; *r*_s_^2^ = 0.084; *p* = 0.001), and verbal memory (*r*_s_ = 0.484; *r*_s_^2^ = 0.234; *p* < 0.0001) (the strongest correlation). There were no significant correlations between polarity of previous episodes and psychomotor speed or verbal fluency scores.

## Discussion

The results obtained in this study come to support the existing evidence regarding neurocognitive deficits in bipolar disorder [2, 20, 21]. The bipolar patients scored lower than the control group in all the cognitive tests applied. Different levels of deterioration were found for verbal memory, working memory, psychomotor speed, verbal fluency, attention and speed of information processing, and executive function/reasoning and problem solving. These findings are not surprising, as previous reports underline existing deficits in areas such as attention [3, 22], verbal and working memory [4, 22–25], and executive function [5], not only during acute episodes but also in euthymia [4, 24, 26–28]. Elshahawi [7] and Chaves [28] emphasized that individuals with bipolar disorder showed consistent impairment of attention. Intact attention capacity is essential to all higher cognitive skills [5]. When compared with patients suffering from schizophrenia, cognitive deficits in bipolar disorder are more circumscribed in nature [29] and involve primary attention processing [30], executive function [31], and verbal memory [32].

Gender differences had no significant impact on cognitive decline. This result is supported by previous data available in the literature—neither Kolur et al. [33] nor Ferrier et al. [34] found any significant gender difference in respect to cognitive function. Due to the fact that patients and control healthy subjects were comparable in age, gender, and education, these factors were most likely not responsible for the differences between groups.

In a study comparing three groups of bipolar patients (manic, depressive, and mixed) and the control group, Basso et al. [35] reported comparable cognitive deficits between the three groups with a significant difference from the control group in the extent of cognitive impairment. However, the scores of all applied tests in the present study showed significant differences among the four assessed subgroups (manic, depressive, euthymic, and mixed).

The manic patients recorded the lowest scores on verbal memory, working memory, and executive function/reasoning and problem solving tests, being in line with the literature. Results obtained by Clark et al. [36] for patients with mania showed deficits in executive function/reasoning and problem solving, McGrath et al. [37] underlined deficits in working memory, and Goldberg and Burdick [5] argued that impairment of verbal memory is present in symptomatic phases as well as in euthymia. Moreover, Clark et al. [36] pointed out that impairment of verbal memory is a core deficit associated with mania. Working memory is necessary for staying focused on a task, blocking out distractions, and keeping persons updated and aware about what’s going on around them [25]. The investigation of cognition in mania is inherently difficult due to the associated distractibility, impulsivity which may have seriously hindered but not totally compromised research of this phase of bipolar illness. Predictably, manic patients seem to have difficulty in concentrating and to be more impulsive when making decisions [25]. The final scores at Token motor task test (which evaluated psychomotor speed) were low due to a higher number of errors made by subjects suffering from mania. Another argument might be that most manic patients received treatment with both benzodiazepines and antipsychotics (typical and atypical) at the moment of cognitive testing; therefore, it is not surprising that they showed a more significant cognitive impairment.

Depressive patients performed less in verbal fluency and psychomotor speed tests. Martinez-Aran et al. [6] and Wolfe et al. [38] concluded that verbal fluency is a cognitive domain specifically affected in depressive patients. In this study, the comparison between the four subgroups showed that depressed patients obtained poorer results in both tests for assessing verbal fluency: ‘category instances’ (semantic fluency) and ‘controlled oral word association test’ (letter fluency). Van der Werf-Eldering et al. [39] confirmed that cognitive dysfunction is more severe in patients with depressive symptoms, particularly psychomotor speed.

Patients with mixed episodes had poor outcomes in tasks associated with working memory, executive function/reasoning, and problem solving, attention, and speed of information processing. Limited data regarding the effects of mixed episodes on cognitive outcomes are available in the literature [40]. In a study comparing mixed/manic patients with depressive bipolar patients, Sweeney et al. [40] showed that patients with mixed episodes displayed impairment of working memory and episodic memory, attention, and executive function/reasoning and problem solving. Previous studies evaluating the memory of manic patients, provided evidence of deficits in working memory [36], [47] with preliminary proofs suggesting that mixed symptoms further exacerbate these deficits [41, 42]. Taking all this into consideration, our results are in concordance with previous published studies.

Euthymic patients achieved significantly higher scores on all tests, compared to the other three subgroups, but significantly lower scores compared to the control group. There is substantial evidence of cognitive deficits in persons who are in remission after acute episodes of bipolar disorder reinforcing the idea that euthymia is not a period of complete recovery [6, 43–46]. As previously mentioned, the patients included in all four subgroups were comparable in terms of age, gender, and education; therefore, we concluded that these factors are less likely to be responsible for the differences between subgroups.

In concordance with the data presented by previous studies [6, 9–12], our results support the correlation between cognitive function and certain characteristics of the course of bipolar disorder (e.g., age at illness onset, illness length, and hospitalizations). For the subgroup of patients with manic episode, a younger age at onset was associated with lower scores on verbal memory test. For those with depressive episode, younger age at onset was associated with lower scores on verbal fluency test. In contrast with our findings, Zubieta et al. [47] and Deckersbach et al. [48] did not find a significant correlation between age of illness onset and cognitive performance.

The length of illness and number of hospitalizations had a negative correlation with verbal memory scores in the manic subgroup, with working memory in the depressive subgroup and with attention and speed of information processing in euthymic patients or patients with mixed episodes. The length of illness was also negatively correlated with psychomotor speed scores in the bipolar group. This finding is consistent with Martinez-Aran’s et al. 's study [24]. Cavanagh et al. [49] and Elshahawi et al. [7] showed that a longer length of illness is correlated with poor performance on verbal memory test. Similarly to the present study, Martinez-Aran et al. [6] observed that the number of hospitalizations had a negative correlation with verbal memory performance. The number of hospital admissions might be considered an indirect measure of severity in terms of individual episodes as well as of course of illness [7].

The assessment of cognitive function in correlation with the polarity of previous mood episodes showed that patients with previous mainly manic episodes had significantly lower scores in tests assessing verbal memory, working memory, attention and speed of information processing, executive function/reasoning, and problem solving compared to their depressed counterparts. Kessing et al. [12], Van Gorp et al. [9], and Martinez-Aran et al. [6] hypothesized that these cognitive deficits might be related to the frequency of episodes, the manic pole having a more extensive impact on neuropsychological function. Previous studies [6, 7, 22, 43, 48, 50] concluded that a higher number of past manic episodes were associated to poorer performance on verbal memory. Ferrier and Thompson [51] argued that the cognitive deficits observed in their study can be confused with the effect of residual symptoms; therefore, cognitive symptoms may actually be among the most sensitive indicators of incomplete remission [50]. An overall understanding of the disease (including the cognitive aspect), its acceptance by the patient, and the caregivers and their adaptability to the disease are major determinants of adherence to treatment and functional recovery [52]. Using neuropsychological evaluations to demonstrate the existence of cognitive deficits is limited by a number of nonspecific factors [52]. Decreased performance on neuropsychological tests may be attributed to the reduction of motivation, poor cooperation, or failure to remain focused during the test, distractibility, hyperactivity, lack of concentration, especially in manic patients.

The study’s limitations comprise the retrospective data gathering regarding the evolution of bipolar disorder (from illness onset to present) which made it difficult for the researchers to collect specific and complete information related to medication history and the periods when the patients were not treated so as to correlate the presence/absence of medication with the cognitive function. This paper represents an intermediate data analysis (cross-sectional assessment of cognitive function and its relationship with previous course of illness) as part of an ongoing study that will perform longitudinal assessment of cognitive function. Additional data will be further published by the authors. Following up this sample, longitudinally, will make us be able to determine the stability or evolution of cognitive impairment that has been stressed by this intermediate cross-sectional analysis.

## Conclusions

The bipolar patients performed worse than healthy controls in verbal memory (list learning), working memory (digit sequencing task), psychomotor speed (token motor task), verbal fluency (category instances—Semantic fluency and controlled oral word association test—letter fluency), attention and speed of information processing (symbol coding), and executive function/reasoning and problem solving (tower of London). Different degrees of cognitive impairment were identified in all phases of the disorder but mainly during manic episodes. A severe course of bipolar disorder (characterized by longer length of illness, younger age of onset, and higher number of hospitalizations) may contribute to the intensity of cognitive deficits. The relevant history for an increased number of manic episodes is correlated with poorer performance on cognitive tests, especially on verbal memory test. Given the specific areas of malfunction in patients with a different predominance of polarity, neurocognitive programs for rehabilitation should be tailored accordingly to the needs of each subgroup of bipolar patients. There is a need for clinical assessment and cognitive tests dynamically applied in order to be able to determine the stability or evolution of cognitive impairment in time.

## Abbreviations

YMRS: Young Mania Rating Scale
HAMD: Hamilton Depression Rating Scale
DSM-IV-TR: Diagnostic and Statistical Manual of Mental Disorders, 4th edition, text revision
